# Miniopen Repair of Ruptured Achilles Tendon in Diabetic Patients

**DOI:** 10.1155/2014/840369

**Published:** 2014-10-30

**Authors:** Abdelsalam Eid

**Affiliations:** Department of Orthopaedic Surgery, Faculty of Medicine, Zagazig University, Zagazig 44511, Egypt

## Abstract

*Background*. Acute degenerative Achilles tendons ruptures may be managed either operatively or nonoperatively with the superiority of the operative treatment in reducing the risk of rerupture. Acute rupture of Achilles tendon is commonly seen in diabetic patients. Open techniques for Achilles tendon repair have been associated with significant complications as deep infection and wound-related problems. *Patients and Methods*. Thirteen type II diabetic patients with acute degenerative rupture of the Achilles tendon were managed by miniopen repair augmented by peroneus brevis tendon. *Results*. All repairs healed successfully. The patients were able to return to preinjury level of activity after a mean of 5 months. The mean ATRS score improved from 15.1 preoperatively to 74.8 postoperatively. The mean Leppilahti ankle score was 59.6. Three patients suffered from superficial wound infection which was successfully managed. However, no patients suffered any major complications such as DVT, deep infection, or reruptures during the period of the study. *Conclusion*. Repair of acute degenerative tear of the Achilles tendon with peroneus brevis tendon augmentation could be successfully performed through a miniopen technique with minimization of wound complications in diabetic patients.

## 1. Introduction

Achilles tendon rupture (ATR) is seen with increasing frequency, often in middle aged individuals [1]. There is no universal agreement on the indications for operative treatment. However, most surgeons agree that surgery is the treatment of choice [2], unless there are contraindications for surgery or if the patient has low functional demands [3].

Both nonoperative and operative treatments have been shown to give good results; however, nonoperative treatment has been associated with a higher rate of rerupture [4, 5].

Therefore, operative treatment may be offered to patients with a relatively higher level of activity aiming for a better outcome and less chances of rerupture. However, operative treatment is not without complications and most of those are wound-related problems [1].

Diabetes has been shown to be associated with pathological changes in the Achilles tendon including rupture [6–8]. In addition, diabetes is associated with an increased susceptibility to wound infections and wound healing problems [9, 10].

Minimally invasive techniques may help minimize wound-related complications in the treatment of Achilles tendon rupture in diabetic patients.

Our hypothesis was that repair of acute degenerative tear of the Achilles tendon with peroneus brevis tendon (PBT) augmentation could be successfully performed through a miniopen technique with minimization of wound complications in diabetic patients.

## 2. Patients and Methods

This prospective study was carried out in the orthopaedic department of our university hospital between January 2008 and June 2011. Thirteen patients with acute degenerative rupture of the Achilles tendon were managed by miniopen repair augmented by peroneus brevis tendon. Nine patients were women. All patients were type II diabetics. The mean age of the patients was 54.8 years (range 47 to 65). The left side was affected in 8 patients. The mean interval between injury and operation was 10.3 days (range 7 to 20 days).

Five patients were housewives, five were office clerks, and three were manual workers. All patients were examined clinically to establish the diagnosis. The Achilles tendon total rupture score (ATRS) [11] was obtained from the patients preoperatively with a mean 15.1. MRI was performed in order to check the status of the proximal segment of the tendon and estimate the expected gap.

All participants gave an informed consent and IRB approval was obtained.

Inclusion criteria were as follows:

1. a degenerative rupture of Achilles tendon;
2. injury within 4 weeks;
3. the degenerative segment less than 5 cm as measured by MRI;
4. the gap that could be closed without tension intraoperatively.

Exclusion criteria were as follows:

1. traumatic tears;
2. injury was older than 4 weeks;
3. the degenerative segment longer than 5 cm;
4. the gap that could not be closed without tension intraoperatively;
5. rerupture of Achilles tendon.

## 3. Surgical Technique (Figure 1)

The patients were given either general or regional anaesthesia. A prophylactic third generation cephalosporin was given. A pneumatic tourniquet was applied. The patients were positioned prone. The affected limb was draped free and the ankle hung over the edge of the table.

Two incisions were made each 3 cm in length. The proximal one was in the middle of the posterior aspect of the calf over the proximal tendon stump (wound 1). The distal one was just lateral to the distal tendon stump (wound 2). It was placed away from the midline to avoid being directly over the tendon repair and causing adhesions. It was placed laterally to be close to the peroneus brevis which will be used for augmentation.

First the proximal stump was delivered through the proximal wound and the degenerative parts of it were removed till healthy tendon tissue was reached. Then a modified Kessler suture was applied to it using nonabsorbable Ethibond number 2 sutures. The distal stump was delivered through the distal wound and debrided. A modified Kessler suture was passed through the remaining distal stump using Ethibond number 2 sutures.

Forceps were passed from wound 2 to wound 1 to grab the suture and deliver the proximal tendon stump into wound 2. An intraoperative assessment of the gap was done at this stage. If the gap could be closed without undue tension with ankle plantar flexion the next step was the harvest of the PBT. If the gap could not be closed the technique was abandoned and a different technique was adopted and the case was excluded from this series.

A two cm incision was then made at the base of the 5th metatarsal to access the PBT (wound 3). The tendon was sharply divided at its end after being secured with a suture. The PBT was then delivered into wound 2. The muscle fibres covering the PBT were gently stripped to clear a length of at least 10 cm of the tendon so that it could be used in the augmentation while preserving the tendon's attachment to its muscle belly.

The two segments of the Achilles tendon were approximated and the modified Kessler suture was tied on both sides. A 15 blade was used to create a horizontal tunnel through the proximal and distal tendon stumps for the passage of peroneus brevis tendon. In 3 cases the distal stump was too small and a drill bit was used to create a tunnel through the calcaneus for the tendon passage.

The PBT was passed first through the distal then through the proximal tunnels and was sutured on itself. Direct absorbable sutures were passed across the Achilles tendon repair and between it and the PBT. The paratenon was repaired using absorbable sutures. The wound was closed a below knee cast was applied with the ankle in equinus.

## 4. Postoperative Care

All the patients received follow-up examination at our orthopaedic department. Follow-up examinations were carried out at 2, 6, 8, and 12th weeks and at every 6 months. The patients were instructed to bear weight on the sound leg using two crutches with nonweight bearing on the operated limb from day 2 postoperatively to decrease the incidence of the thromboembolic complications. The patient received intravenous antibiotics for 2 days postoperatively then another 5 days on oral antibiotics. Low molecular weight heparin was given prophylactically for five weeks to guard against DVT. The sutures were removed after 2 weeks. A cast was applied in the plantigrade position of the ankle at 4 weeks postoperatively. Weight bearing in the cast was allowed as tolerated by the patients with the aid of one crutch in the contralateral side. After 8 weeks, the cast was removed and the patient was allowed partial weight bearing with two crutches with a shoe-lift which graduated to full weight bearing over the next month.

Rehabilitation also included an exercise program in the form of strengthening exercises for leg muscles, increasing ankle joint range of motion, and balancing exercises. These exercises increased in strength gradually for the next 3 months [12].

## 5. Follow-Up

Follow-up evaluation was performed at 6 months postoperatively and then yearly thereafter. The mean follow-up was 25.2 (range 18–36 months).

## 6. Results

All repairs healed successfully. This was judged by the disappearance of pain and limp, the ability to stand on tip toe, and the disappearance of the palpable gap in the tendon noted preoperatively. The patients were able to return to preinjury level of activity after a mean of 5 months (range 4–6).

Visual analogue scale (VAS) was used to measure the pain during weight bearing (10 = severe pain and 0 = no pain). Pain during weight bearing was assessed by the VAS which improved from a mean of 8.2 (range: 6 to 10) preoperatively to a mean of 1.6 (range: 0 to 2) at the last follow-up.

Functional outcome was evaluated using the ATRS [11] score as well as the single leg heel rise test. The single leg heel rise test was performed according to the instructions of Nilsson-Helander et al. [12] The healthy side was examined first and the number of heel rises per minute was recorded and compared to the other side. At the last follow-up, the mean number of heel rises per minute of the operated side was 29 (range: 25 to 32) which was comparable to the nonoperated side with a mean of 32.5 (range: 29 to 35). The mean ATRS score improved from 15.1 preoperatively (range: 11 to 22) to 74.8 postoperatively (range: 68 to 83).

Patients' subjective assessment of the functional outcome was evaluated using the modified Leppilahti ankle score (max. 70 points) [1]. The mean Leppilahti ankle score was 59.6 (range 55 to 65).

## 7. Complications

Three patients (23%) suffered from superficial wound infection and they were treated by repeated dressing and antibiotics according to wound swab culture and sensitivity (Table 1). None of our patients suffered any major complications such as DVT or deep infection. There were no reruptures during the period of the study.

## 8. Discussion

Acute rupture of Achilles tendon is seen with increasing frequency [13]. Acute degenerative Achilles tendons ruptures may be managed either operatively or nonoperatively with the superiority of the operative treatment in reducing the risk of rerupture [2, 5, 13]. The musculoskeletal complications in the diabetic patients are common and one of these is the ATR [7, 8]. In addition, diabetes is notorious for postoperative complications especially wound infection and delayed healing [9, 10]. Deep infection after Achilles tendon repair is a significant complication that can require split-thickness skin grafting or flap coverage and may result in complete loss of the Achilles tendon [13]. The miniopen technique can be beneficial in these circumstances as the preservation of skin cover during reconstruction procedures helps protect the reconstruction beneath [2]. The use of peroneus brevis tendon with preservation of its blood supply can augment the repair and reduce the risk of the rerupture.

Nilsson-Helander et al. [12] compared conservative to operative treatment in 100 patients divided into two groups. Using a classic open repair technique in their operatively treated group they reported a mean ATRS of 88 points after 12 months postoperatively. However, in their operatively treated group they also reported a 4% rerupture rate, one case of Achilles tendon contracture, a 34% occurrence of DVT detected by colour duplex sonography, two infections, 1 deep and 1 superficial, and finally, thirteen patients (26%) with complaints concerning the scar.

Maffulli et al. [8] operated on 39 diabetic cases using a percutaneous technique. They reported a mean ATRS score of 70.4. However, they had a superficial wound infection rate of about 20.5% (8 patients) and deep wound infection of about 28.2% (11 patients), two of them needed surgical debridement.

The results of this series are comparable to those other authors in terms of pain relief and improvement of function with fewer complications. Having fewer complications than Nilsson-Helander et al. [12] may be attributed to the fact that they used a classic open technique with understandably more potential for wound complications.

As for Maffulli et al. [8], the smaller numbers of patients in this series may account for the limited appearance of complications as compared to a larger series.

In this study all patients were diabetic. All patients were able to return to their preoperative level of physical activities at a mean of 5 months postoperatively. The mean number of heel rises per minute of the operated side was comparable to the nonoperated side. Only three patients suffered from superficial wound infection and they were successfully managed conservatively. None of the patients suffered any major complications or reruptures during the period of the study. This supports our hypothesis that repair of acute degenerative tear of the Achilles tendon with peroneus brevis tendon augmentation could be successfully performed through a miniopen technique with minimization of wound complications.

Limitations of this study include the small number of patients and lack of a control group. This should be addressed in future work.

## 9. Conclusion

Repair of acute degenerative tear of the Achilles tendon with peroneus brevis tendon augmentation could be successfully performed through a miniopen technique with minimization of wound complications in diabetic patients.

## Figures and Tables

**Figure 1:**
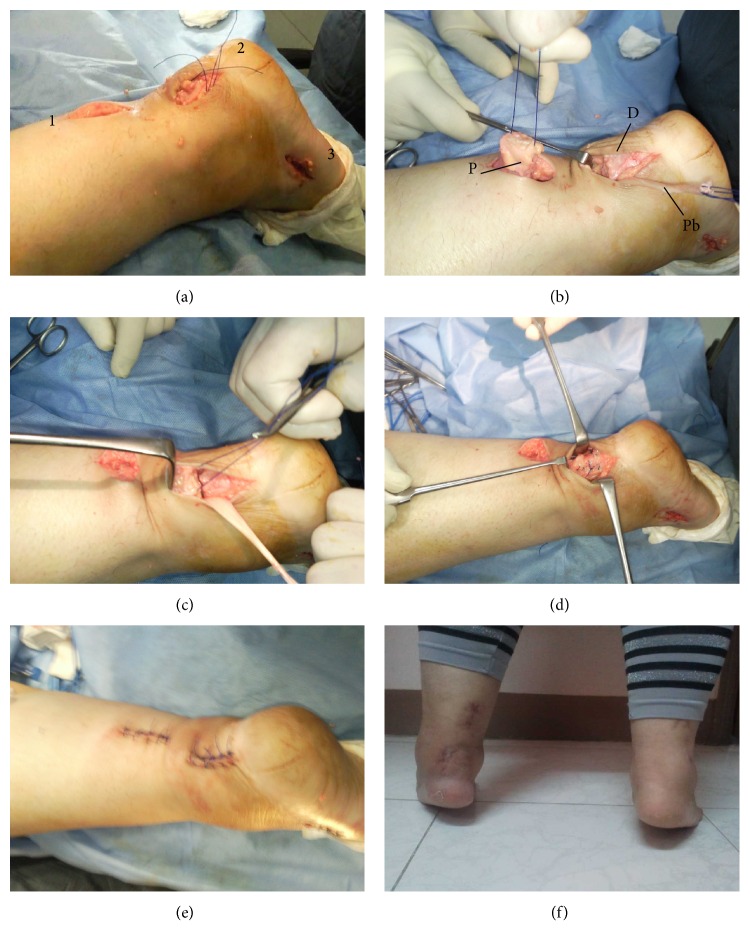
Surgical technique and functional result. (a) The three incisions (1, 2, 3). (b) The proximal stump (P), the distal stump (D), the peroneus brevis (Pb). (c) The proximal stump has been delivered into wound 2 and the gap is closed. (d) The repair is done and augmented by (Pb). (e) Skin closure. (f) Functional result.

**Table 1:** Summary of the patients' data.

Number	Age	Sex	Pre-op VAS	Post-op VAS	Heel rises operated	Heel rises healthy	ATRS Pre-op	ATRS final	Modified Leppilahti	Complications
1	47	F	9	2	25	29	18	75	60	
2	65	F	9	1	27	32	13	75	55	No
3	56	M	8	2	31	35	10	83	65	No
4	63	F	7	1	32	35	16	70	55	No
5	49	M	8	0	30	34	17	83	60	No
6	52	F	9	0	27	31	22	70	60	Superficial infection
7	61	F	10	2	29	30	14	73	65	No
8	56	F	6	1	30	34	17	78	60	Superficial infection
9	47	M	7	0	32	35	15	76	65	No
10	49	F	7	2	26	30	19	68	55	No
11	55	F	9	2	29	31	13	76	55	No
12	53	M	9	2	28	32	12	77	60	No
13	60	F	9	1	31	34	11	68	60	Superficial infection

Mean			8.2	1.2	29.0	32.5	15.2	74.8	59.61538	
